# A major impact of the influenza seasonal epidemic on intensive care units, Réunion, April to August 2016

**DOI:** 10.2807/1560-7917.ES.2016.21.47.30405

**Published:** 2016-11-24

**Authors:** Laurent Filleul, Dany Bakoly Ranoaritiana, Elsa Balleydier, David Vandroux, Clémence Ferlay, Marie-Christine Jaffar-Bandjee, Julien Jaubert, Bénédicte Roquebert, Bruno Lina, Martine Valette, Bruno Hubert, Sophie Larrieu, Elise Brottet

**Affiliations:** 1 Santé publique France, French national public health agency, Regional unit (Cire) Océan Indien, Réunion, France; 2 Indian Ocean Field Epidemiology Training Programme, Surveillance des Epidémies et Gestion des Alertes (SEGA) One Health Network, Indian Ocean Commission, Mauritius; 3 Epidemiological Surveillance Department, Ministry of Health, Madagascar; 4 Intensive Care Unit, Centre Hospitalier Universitaire, Saint-Denis, Réunion, France; 5 Intensive Care Unit, Centre Hospitalier Universitaire, Saint-Pierre, Réunion, France; 6 Laboratory of virology, Centre Hospitalier Universitaire, Saint-Denis, Réunion, France; 7 Laboratory of biology, Centre Hospitalier Universitaire, Saint-Pierre, Réunion, France; 8 Hospices Civils de Lyon, National Influenza Centre, Laboratory of Virology & Virpath, CIRI, Inserm U1111, CNRS UMR5308, ENS Lyon, UCBL, Lyon, France; 9 Santé publique France, French national public health agency, Regional unit (Cire) Pays de la Loire, Nantes, France

**Keywords:** viral infections, influenza-like illness - ILI

## Abstract

The 2016 seasonal influenza in Réunion in the southern hemisphere, was dominated by influenza A(H1N1)pdm09 (possibly genogroup 6B.1). An estimated 100,500 patients with acute respiratory infection (ARI) consulted a physician (cumulative attack rate 11.9%). Sixty-six laboratory-confirmed cases (65.7/100,000 ARI consultations) were hospitalised in an intensive care unit, the highest number since 2009. Impact on intensive care units was major. Correlation between severe cases was 0.83 between Réunion and France and good for 2009 to 2015.

Réunion is a southern hemisphere French overseas territory with 843,529 inhabitants (2015 estimate [1]) located in the Indian Ocean between Madagascar and Mauritius. The island benefits from a healthcare system similar to mainland France. In the 2016 influenza season lasting from April to August, Réunion experienced a high number of severe influenza cases.

## Influenza surveillance system and definition of severe cases

Influenza is monitored through a multi-source surveillance system including a sentinel general practitioners (GPs) network, hospital emergency departments, intensive care units (ICUs), laboratory and mortality data [2]. The sentinel GPs network [3] is based on reports from 53 volunteer GPs located throughout the island. They report on weekly basis to the regional office of the French national public health agency (Cire OI) their total number of consultations and number of consultations for acute respiratory infections (ARI) (defined as a sudden onset of fever (≥ 38 °C) and cough, which are associated or not with other symptoms, such as for example breathing difficulty or headache). In addition to the weekly proportion of ARI among sentinel consultations, a weekly estimated number of ARI consultations is extrapolated from the total number of consultations in Réunion which are derived from health insurance data. Severe cases of influenza are reported in real-time by clinicians of ICUs to the Cire OI. A severe influenza case is defined as a patient with laboratory-confirmed influenza (positive RT-PCR for influenza virus) admitted for more than 24 hours to an ICU.

## The 2016 influenza epidemic in Réunion

**Figure 1:**
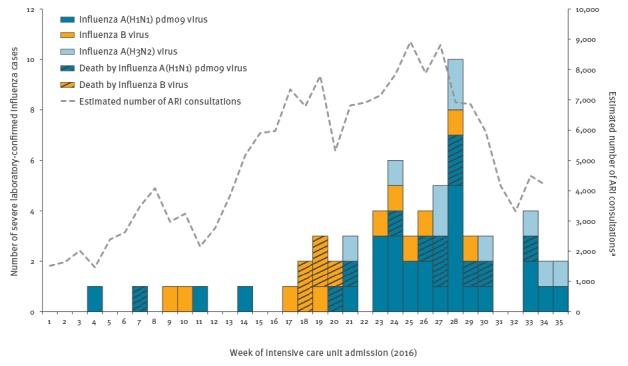
Severe influenza cases by virus type and death, Réunion, France, week 1 to week 35, 2016 (n = 66)

In 2016, the influenza epidemic period in Réunion started one month earlier than usual (week 17, end of April) and ended in week 30 (Figure 1). The epidemic peak was reached at week 27 in July. During that week, the estimated number of consultations due to ARI was 8,700. Over the whole epidemic period, the number of patients with ARI who consulted a GP was estimated at 100,500 which represents a cumulative attack rate of 11.9% (100,585 / 843,529) in the general population.

ARI: acute respiratory infections.

^a^ Extrapolated from the total number of consultations in Réunion, which were derived from health insurance data.

At the beginning of the epidemic period, we observed mainly influenza B virus circulation, and after 6 weeks, influenza A(H1N1)pdm09 virus became the predominantly circulating virus on the Island. We also detected some A(H3N2) viruses but they accounted for only 20% of influenza viruses identified through surveillance. Influenza B virus strains were those targeted by the 2016 seasonal vaccine for the southern hemisphere (B/Victoria) [4].

Between January and August 2016, 66 laboratory-confirmed influenza cases with severe disease were identified: 15 (23%) were infected with influenza B virus, 11 (17%) with A(H3N2) and 40 (61%) with A(H1N1)pdm09. The first virological analyses from the French national influenza reference centre in Lyon, France (sequencing ongoing), identified A(H1N1)pdm09 possibly related to genogroup 6B.1 in eight cases from surveillance and in seven severe cases infected by influenza A(H1N1)pdm09 virus.

The incidence rate of severe cases over the whole season was 65.7 per 100,000 ARI consultations in 2016, higher (1.5 times) than that observed in 2014 (46.0/100,000), and the highest observed since the start of surveillance in 2009 [5]. When only the epidemic period was considered, the incidence in 2016 was 51.7 per 100,000 vs 31.7 per 100,000 in 2014.

**Figure 2:**
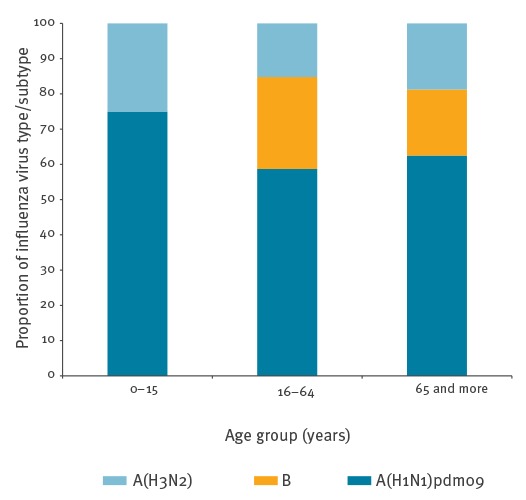
Severe influenza cases by age group and virus type/subtype, Réunion, France, week 1 to week 35, 2016 (n = 66)

Median age of the 66 severe cases was 53.5 years (range: one month to 86 years). We did not observe any trend in the distribution of influenza virus types according to age among severe cases (Figure 2), nevertheless, the majority of cases were aged over 41 years (52/66) irrespective of the incriminated viruses. Sex ratio (M/F) was 1.27 (37/29).

Medical characteristics of patients are presented in Table. Among the 66 cases, 46 (70%) required mechanical ventilation, and of them 36 presented signs and symptoms compatible with criteria for acute respiratory distress syndrome (ARDS) using the Berlin ARDS definition [5]. Eight of 36 needed extracorporeal membrane oxygenation (ECMO). The case fatality ratio was 27%, 18 of 66 patients died. Median of Simplified Acute Physiology Score II (SAPS II) score was 47.6 (range: 16–101). Regarding risk factors (Table), 60 cases had risk factors including chronic respiratory disease (n=28), age ≥ 65 years (n=16) and diabetes (n=15). Of 58 severe cases where the vaccination status was known, 53 were unvaccinated.

**Table:** Characteristics of severe influenza cases, Réunion, influenza season 2016 (n = 66)

Influenza virus types/subtypes	A(H1N1)pdm09 (n = 40)	B (n = 15)	A(H3N2) (n = 11)
**Sex** (Male / Female)	25/15	8/7	4/7
**Median age** in years (range)	54.5 (0–76)	55 (21–86)	48 (13–76)
**Risk factors**			
Age ≥ 65 years	10	3	3
Age < 1 year	2	0	0
Chronic respiratory disease	15	5	8
Diabetes	9	3	3
Cardiac disease	5	2	0
Neuromuscular disease	3	2	0
Obesity (Body mass index > 30)	6	2	0
Pregnancy	2	1	1
Hepatic disease	0	2	0
Immunodeficiency	3	0	0
None	2	3	1
**Indicators of signs of severity**			
Median Simplified Acute Physiology Score II (SAPS II) (range)	37.5 (16–95)	46.0 (17–101)	45.0 (21–65)
Respiratory assistance:	25	12	9
*- with acute respiratory distress syndrome (ARDS)*	*19*	*9*	*8*
*- with ARDS needed extracorporeal membrane oxygenation (ECMO)*	*5*	*1*	*2*
**Death**	13	5	0
**Influenza vaccination**			
Unvaccinated	34	11	8
Vaccinated	2	1	2
Not specified	4	3	1

## Correlation between number of severe influenza cases in Réunion and mainland France

**Figure 3:**
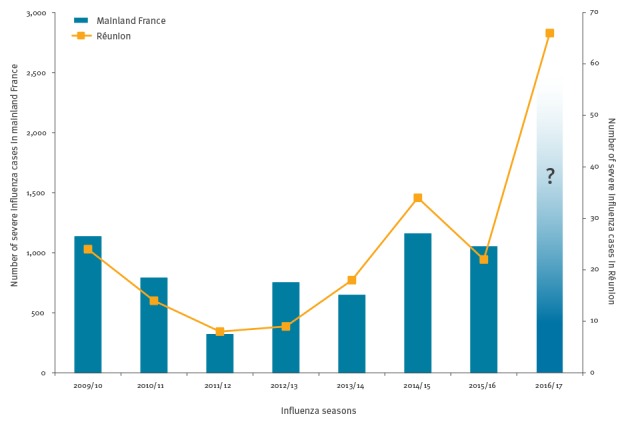
Number of severe influenza cases in mainland France and in Réunion by influenza seasons, 2009–2016

When we compared trends in the number of severe cases in Réunion and mainland France using data from the national influenza surveillance system over the past influenza seasons, we observed a good correlation between them [6]. During the years 2009 to 2015, regardless of circulating virus types or subtypes, the Pearson’s correlation coefficient between number of severe cases in Réunion and mainland France was 0.83. For each increase in the number of cases in ICU observed in Réunion, the next season in mainland France was also characterised by an increase in severe influenza cases (Figure 3).

Influenza seasons labelled for the northern hemisphere. The corresponding southern hemisphere seasons are 2009, 2010, 2011 etc.

The northern hemisphere influenza season has started only recently and numbers of severe cases are not yet available. If a similar situation to that in Réunion happened during the 2016/17 influenza season in mainland France and potentially other European countries, we might observe an increase of severe influenza cases.

## Discussion

The 2016 influenza epidemic period on Réunion was characterised by an unusual duration of 14 weeks compared to a mean of 8 weeks in previous years [7]. Severe cases in ICUs were mainly related to influenza A(H1N1)pdm09 virus infections. Compared with 2014, we observed twice the number of severe influenza cases in 2016 and it was three times that of other previous years. However, we did not observe an increased case fatality ratio compared with previous years.

Individual factors did not allow us to infer causes for this high number of cases, since we found common risk factors for influenza such as chronic respiratory disease, diabetes, cardiac disease or age. In this respect, we did not observe any significant differences between previous seasons or type/subtype of viruses [7].

The characterisation of circulating viruses showed that influenza B and influenza A(N1N1)pdm09 viruses were similar to the strains included in the 2016 southern hemisphere seasonal influenza vaccine, used in Réunion [4]. Worldwide, two genetic subclades of viruses within the 6B clade have emerged, designated as subclades: 6B.1 defined by HA1 amino acid substitutions S162N and I216T and 6B.2 defined by HA1 amino acid substitutions V152T and V173I [8]. Chambers et al. showed that the vaccine provided significant protection against A(H1N1)pdm09 illness despite genetic evolution in circulating viruses [9].

The influenza immunisation coverage among the target population (age >65 years old, chronic diseases, pregnant women) is low in Réunion (around 34% in 2016), and this was confirmed by our data where a minimum of 53 severe cases were not vaccinated and 60 cases had risk factors. While the low immunisation coverage could explain the severity of the outbreak, it is not sufficient to explain the unusual number of severe cases since immunisation coverage was already low during the past few years.

Our data showed a major impact on public health of the 2016 influenza epidemic in terms of influenza-related morbidity and incidence of severe cases requiring treatment in ICUs, but not for case fatality [7]. The demonstrated correlation between severity of cases in different seasons in Réunion and mainland France is based on the data observed and not the result of a modelling exercise. This fact should be taken in consideration. Future studies should confirm the pattern and the conclusions that can be drawn from the impact of influenza seasons on ICUs in Réunion for the situation in the following influenza season in France.

If a similar situation to that in Réunion happened during the 2016/17 influenza season in mainland France and potentially other European countries, we might observe an increase of severe influenza cases. This information can be useful to strengthen prevention i.e. by improving immunisation coverage for the 2016/17 season and to prepare ICUs to be able to care for possibly more influenza patients than usual.
